# Transcriptional response of mushrooms to artificial sun exposure

**DOI:** 10.1002/ece3.7862

**Published:** 2021-07-05

**Authors:** Franz‐Sebastian Krah, Jaqueline Hess, Florian Hennicke, Ritwika Kar, Claus Bässler

**Affiliations:** ^1^ Conservation Biology Institute for Ecology, Evolution and Diversity Faculty of Biological Sciences Goethe University Frankfurt Frankfurt am Main Germany; ^2^ Department of Soil Ecology UFZ Helmholtz Centre for Environmental Research Halle (Saale) Germany; ^3^ Project Group Genetics and Genomics of Fungi Chair Evolution of Plants and Fungi Ruhr‐University Bochum (RUB) Bochum Germany; ^4^ Centre for Plant Molecular Biology, Developmental Genetics University of Tübingen Tübingen Germany; ^5^ Bavarian Forest National Park Grafenau Germany

**Keywords:** climate change, experiment, fungi, heat, heat‐shock protein, light, mushroom, sun exposure, transcriptome

## Abstract

Climate change causes increased tree mortality leading to canopy loss and thus sun‐exposed forest floors. Sun exposure creates extreme temperatures and radiation, with potentially more drastic effects on forest organisms than the current increase in mean temperature. Such conditions might potentially negatively affect the maturation of mushrooms of forest fungi. A failure of reaching maturation would mean no sexual spore release and, thus, entail a loss of genetic diversity. However, we currently have a limited understanding of the quality and quantity of mushroom‐specific molecular responses caused by sun exposure. Thus, to understand the short‐term responses toward enhanced sun exposure, we exposed mushrooms of the wood‐inhabiting forest species *Lentinula edodes,* while still attached to their mycelium and substrate, to artificial solar light (ca. 30°C and 100,000 lux) for 5, 30, and 60 min. We found significant differentially expressed genes at 30 and 60 min. Eukaryotic Orthologous Groups (KOG) class enrichment pointed to defense mechanisms. The 20 most significant differentially expressed genes showed the expression of heat‐shock proteins, an important family of proteins under heat stress. Although preliminary, our results suggest mushroom‐specific molecular responses to tolerate enhanced sun exposure as expected under climate change. Whether mushroom‐specific molecular responses are able to maintain fungal fitness under opening forest canopies remains to be tested.

## Graphical abstract

We experimentally study mushroom‐specific effects of artificial sun exposure. Although preliminary, our results point toward molecular responses of mushrooms to cope with sun exposure, suggesting coping mechanisms are active to respond to climate change.

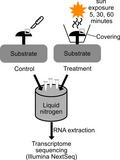

## 1 INTRODUCTION

Forests have undergone increasing tree mortality associated with hot drought episodes, wind storms, insect outbreaks, and fires, and tree mortality is likely to increase further under climate change (Seidl et al., 2017; Senf et al., 2018). Tree die‐off leads to an increase of forest gaps and consequently to increased sun exposure to the forest floor due to reduced canopy. Whereas forest floors under closed canopies are characterized by benign temperatures and buffering of extremes, open canopies lead to short‐term higher variability of temperatures and radiation and thus to extremes (Frenne et al., 2019; Kermavnar et al., 2020; Scharenbroch & Bockheim, 2007). Effects of microclimates associated with open canopies can even exceed the effects of the current increase of mean climate values (Sher et al., 2004; Zellweger et al., 2020). Changes in microclimates due to canopy variability have been shown to affect many forest organisms such as beetles, plants, and fungi (Krah et al., 2018; Müller et al., 2020; Seibold et al., 2016; Zellweger et al., 2020). The ability of an organism to adapt to a variety of environmental conditions defines its spatial and temporal prevalence. The distributional boundaries are often explained by stressful environmental conditions. Stressful environmental conditions are defined as leading to a fitness reduction (Hoffmann & Parsons, 1991). To maintain fitness in stressful environments, organisms can adapt and respond physiologically and biochemically (Anjum et al., 2011) or have to disperse or extirpate. Understanding these responses and the underlying mechanisms is essential to predict the potential to adapt to changing environmental conditions of organisms, for example, increasing sun exposure caused by canopy loss. In fungi, effects of temperature or radiation have so far been primarily studied for fungal mycelia and fruiting induction (Al‐Obaidi, 2016; Kües & Liu, 2000). However, the potential of grown fruiting bodies, the sexual reproduction organs of many fungi, to respond physiologically and biochemically to environmental stress like sun exposure is currently largely underexplored.

Mushroom‐forming fungi are a species‐rich group with ca. 36,000 species currently described within the Agaricomycetes (Sánchez‐García et al., 2020) and consist of belowground mycelium and aboveground soft‐fleshed fruiting bodies (hereafter also referred to as “mushrooms”). Mushrooms are the organ of reproduction, producing and releasing sexual spores. Hence, they are the source of genetic diversity (Lee et al., 2010). Prior to maturation, fruiting cues start the fructification process by the formation of so‐called “fruiting body initials,” then primordia, then maturating fruiting bodies, and finally mature fruiting bodies with sexual spores formed (Kües & Liu, 2000). Mushroom‐forming fungi are sessile and can reach novel habitats mainly through spore dispersal. The mushroom protects spore development, for example, from radiation via pigmentation to avoid damage of the spore genomes (Cordero & Casadevall, 2017; Halbwachs et al., 2016). For mushroom‐forming fungi, successful fruiting, reproduction, and spore development are, therefore, the basis of successful dispersal, maintaining existing and establishing new populations. These processes are, therefore, crucial for population dynamics. Considering the importance within the fungal life cycle, damage of the mushroom would prevent dispersal and, across longer time periods, the establishment of new fungal mycelium. Such damage might be caused by harsh microclimatic conditions, for example, as present under open canopies. Further, fungi adapted to deadwood as a resource are restricted to produce fruit bodies within the environment where the deadwood is situated. The environment surrounding deadwoods can range from dense conditions (e.g., single tree die‐off) to larger openings caused by disturbance events (e.g., windthrow; Seidl et al., 2017). Due to the passive exposure of mushrooms to environmental conditions, molecular responses can be expected to ensure mushroom integrity. However, molecular capabilities of fungi via the mushroom have been rarely studied compared to mycelium (Halbwachs et al., 2016). Mycelial responses to the abiotic environment are well studied, for example, in studies using transcriptomes (Erdmann et al., 2012; Fu et al., 2016; Wang et al., 2013), proteomes (Liu et al., 2017; Tang et al., 2016), or physiology (Anke & Schüffler, 2018). In contrast, mushroom‐specific responses to (micro)climate have rarely been studied in mushrooms. Also, given the differing molecular biology of mushrooms during fructification (Orban et al., 2021), and their more direct exposure to abiotic stress, knowledge based on the mycelium seems of limited significance for understanding how mushrooms would react.

Identifying differentially expressed genes (DEGs) as a response to sun exposure allows the first understanding of mushroom‐centered regulation of physiological responses (Gracey, 2007). Previous studies investigating mushroom transcriptomics have focused on diverse topics, including conserved developmental genes, regulators, and tissue specificity (Choi et al., 2006; Sakamoto et al., 2017; Song et al., 2018). Although a study tested the effect of temperature treatment on *Flammulina* mushrooms applying 18°C versus 10°C, these temperatures reflect indoor culturing conditions rather than ecologically realistic temperatures under open canopies (Liu et al., 2020; Müller et al., 2020). Other studies investigated the effect of heat on edible mushroom species; however, they applied the treatment to the whole fungal organism (Lu et al., 2014) or harvested the mushroom prior to transcriptomic analysis (Sakamoto et al., 2017), which simultaneously leads to rapid water loss (drought) rather than effects of temperature alone. Thus, the mushroom‐specific molecular response to sun exposure, independent from the mycelium, is currently unknown, preventing predictions, for example, of mushroom performance under increased canopy loss.

**FIGURE 1:**
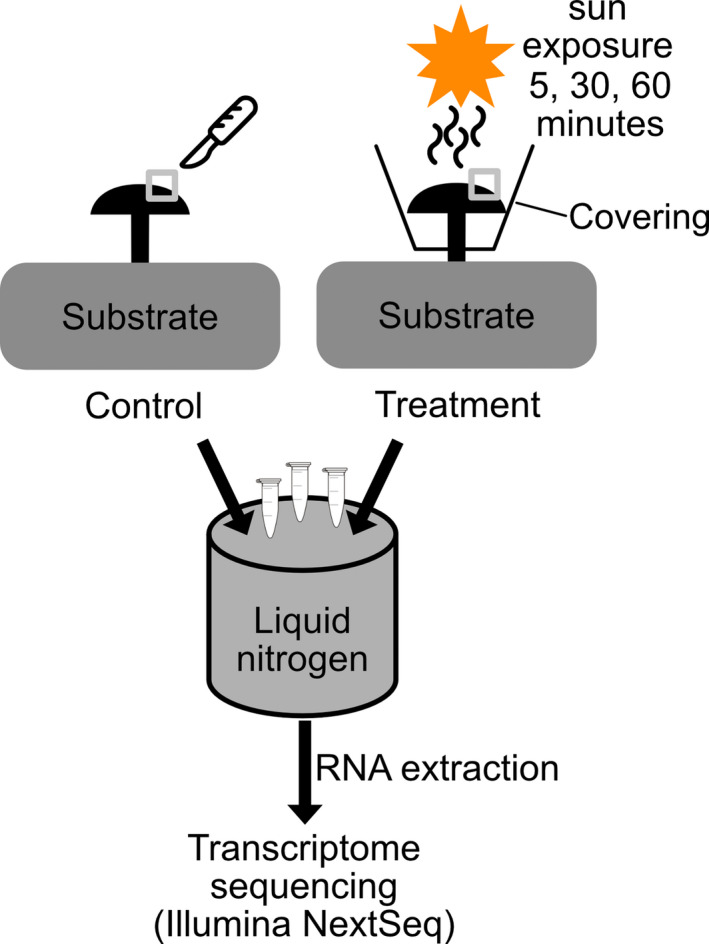
Experimental design of sun exposure using artificial sunlight. Cap surface samples from the mushroom caps of *Lentinula edodes* were taken before treatments (left side) and after the treatments (right side). Mushrooms were subjected to artificial sun exposure from a distance of 10 cm, creating a cap surface temperature of ca. 30°C. We exposed the mushroom cap for 5, 30, and 60 min and then sampled ca. 1 cm^3^ piece, which we directly transferred into liquid nitrogen for further processing (RNA extraction, sequencing). The temperature was measured using an infrared thermometer. We used a total of four controls and three replicates per time point

This study asks whether mushrooms, after successful fruiting cues, display specific molecular responses to cope with short‐term sun exposure, reflecting heat and light stress. We experimentally applied artificial solar light to the Shiitake mushroom (*Lentinula edodes* (Berk.) Pegler) while still being attached to the mycelium (Figure 1). The mushroom was covered to prevent sun exposure of the mycelium. *L. edodes* is a popular edible mushroom (Chang, 1999; Stamets, 2011) and a model organism in many mushroom transcriptome studies (Chen et al., 2016; Choi et al., 2006; Sakamoto et al., 2017; Song et al., 2018). The species *L. edodes* is a forest fungus growing on deadwood and is thus potentially affected by increasing canopy openness and associated sun exposure. To acquire a deeper understanding of molecular responses by mushrooms to sun exposure, we apply transcriptome analysis. We addressed two research questions: (a) Do mature mushrooms, exposed to short‐term artificial sun exposure, differ in their transcriptome compared to the control? And (bii) is the transcription of temperature‐related molecular responses (i.e., heat‐shock proteins) increased compared to the control?

## 2 METHODS

### 2.1 Mushroom material

We ordered two pre‐inoculated substrate blocks with *L. edodes* (https://www.pilzmaennchen.de) inoculated from the same genet. We kept the substrate blocks (hereafter “block”) under moist conditions and ca. 20°C until mushrooms reached maturity (7 days). Maturity was determined by monitoring the time point of spore release and verified whether spores reached values from the literature (Knudsen & Vesterholt, 2008). The two blocks were kept under the same conditions during the whole experiment.

### 2.2 Experimental design and sampling

To mimic sun exposure, we placed caps beneath a solar lamp (Bright Sun UV Desert, 50 W, Lucky Reptile, Germany), which we positioned at a distance of ca. 10 cm from the mushroom cap. According to manufacturer information, this is equal to ca. 60°C and ca. 396,000 lux in 10 cm distance to the lamp. In 20 cm distance, this would drop to ca. 45°C and 99,000 lux. We, however, measured ca. 30°C on the cap surface after 5 min with an infrared thermometer. In comparison, a sunny summer day is characterized by ca. 100,000 lux (Li et al., 2005), and for example, *Pleurotus ostreatus* did not produce fruiting bodies above 4,600 lux (Arjona et al., 2009), implying that 100,000 lux is a stressful condition.

A plant growth pot further surrounded the mushrooms to avoid substrate (mycelium) heating (Figures 1 and S1). Therefore, single mushrooms were exposed to heating for 0 (Control), 5, 30, or 60 min prior to tissue sampling. We used a total of 3 replicates per time point (5, 30, and 60 min) and four controls. The 5‐ and 60‐min samples were taken from block one and the 30‐min samples from block two. Each sample was taken from a different mushroom to avoid effects of prior sampling on the transcriptome, for example, via defense responses. A total of 13 samples were taken for transcriptome analysis. One block did not spawn enough mushrooms, and thus, a second fruiting block was used at the same time. Samples were taken with a scalpel, which was cleaned with 75% ethanol between each sample. Using the scalpel, we cut a ca. 1 cm^3^ piece from the mushroom cap surface and placed it into an Eppendorf tube, which was directly transferred into liquid nitrogen for further processing (Figure 1).

### 2.3 RNA extraction and sequencing and data processing

The frozen fungal tissues were homogenized in a precooled (liquid nitrogen) bead‐mill (Retsch MM 200) with stainless steel beads (2 beads of 3 mm) at high speed (20–30 Hz) for two minutes, and the step was repeated twice for complete homogenization. The total RNA was purified from ca. 100 mg of the frozen homogenized fungal fruiting bodies according to the Qiagen RNeasy Mini kit instructions (Protocol: Purification of Total RNA from Plant Cells and Tissues and Filamentous Fungi). The quality of the purified RNA was estimated on 1% agarose gel electrophoresis and 260/280 nm absorbance value ratio using Epoch Microplate Spectrophotometer (BioTek; Table S1; Figure S2).

Illumina's TruSeq stranded RNA library preparation kit, including poly‐A enrichment, was used to construct libraries from total RNA. Subsequently, the Illumina NextSeq 500/550 platform and a v2 kit (75 cycles total) were used to sequence the libraries. The resulting single‐end reads that passed Illumina's chastity filter were subject to demultiplexing and trimming of Illumina adaptor residuals using Illumina's bcl2fastq software version 2.19.1.403 (no further refinement or selection). The quality of the reads in fastq format was checked with the software FastQC version 0.11.7. The splice‐aware RNA mapping software STAR version 2.6.1d was used to map the reads to the reference genome of *L. edodes* of strain W1‐26 (Chen et al., 2016). To count the uniquely mapped reads to annotated genes, we used the software htseq‐count version 0.11.0 (Anders et al., 2015). Normalization of the raw counts and differential gene expression analysis was carried out with the help of the R software package DESeq2 version 1.18.1 (Love et al., 2014). Differentially expressed genes (DEGs) between the different exposure times of artificial light and control with zero time exposure were analyzed using DESeq2 (Love et al., 2014) with a log_2_‐fold change threshold of 0.5 and alpha level of 0.05. Libraries, sequencing, and data analysis described in this section were performed by Microsynth AG (Balgach, Switzerland).

### 2.4 Annotation and functional enrichment analysis

The predicted protein set (version 1.0) of *L. edodes* strain W1‐26 was downloaded from the Shitake Genome Database (http://legdb.chenlianfu.com/) in March 2019. Updated functional annotations were produced with InterProScan 5 (Jones et al., 2014), using all available search tools. To complement domain‐based annotations, EggNOG‐Mapper version 1.0.3 (Huerta‐Cepas et al., 2017) was used to screen the protein set in one2one ortholog mode and limiting the taxonomic scope to fungi. KOG (euKaryotic Orthologous Groups) annotations were parsed from eggNOG results, and the KOGMWU R package (Dixon et al., 2015) was used to calculate the difference in ranks (delta rank) for each KOG category to visualize relative increase or decrease in expression for each category and time point. KOG categories with significantly different delta ranks among time points were determined using a Mann–Whitney *U* test and corrected for multiple testing using the false discovery rate (FDR), setting alpha = 0.05.

## 3 RESULTS

In total, 424,820,821 clean sequence single‐end reads were obtained from the RNA extracted from mature mushrooms, with a *Q*20 percentage >97% and a mean *Q* of 35. Trimming data resulted in an average read length of 75 bp across all samples. Reads per library were very consistent with an average of 32,678,525 and overall high alignment percentages >92% (Table S2).

**FIGURE 2:**
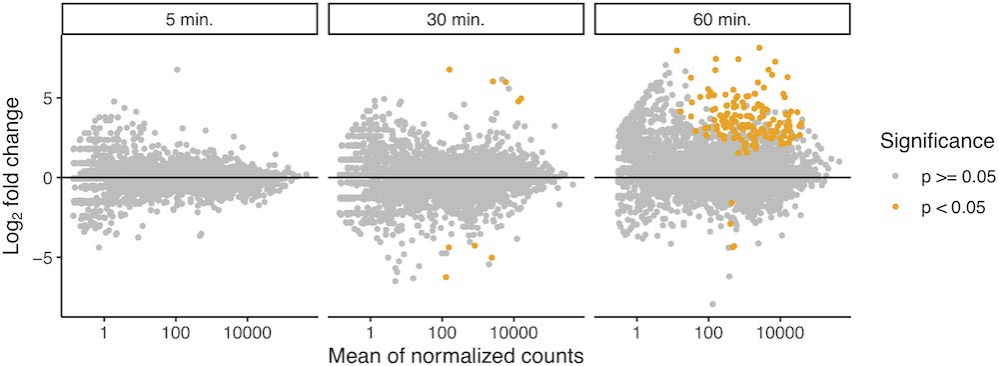
Summary of the differentially expressed genes (DEGs) between the time points (5, 30, and 60 min of artificial sun exposure (ca. 30°C, ca. 100,000 lux)) and control (0 min) of mature fruiting bodies of *L. edodes*. MA plot showing the means of expression signal (*x*‐axis) and log_2_‐fold changes (*y*‐axis) of differential expression (adjusted *p*‐values) during different temporal stages, based on normalized RNA‐seq read counts. Each point indicates a transcript, with non‐significant differential expression in gray and those showing significant differential expression in orange. Significance was estimated using Wald tests, and *p*‐values were adjusted using the false discovery method (Benjamini & Hochberg, 1995) and an alpha level of 0.05

We found no significant up‐ or downregulated DEGs in the 5‐min treatment compared with the control. However, we found significant DEGs at 30 min and a higher number of DEGs at 60 min compared to control (Figure 2). Of 12,069 genes, nine were significantly differentially expressed at 30 min and 148 at 60 min. Out of the 148 DEGs at 60 min, 144 (97%) were upregulated.

**FIGURE 3:**
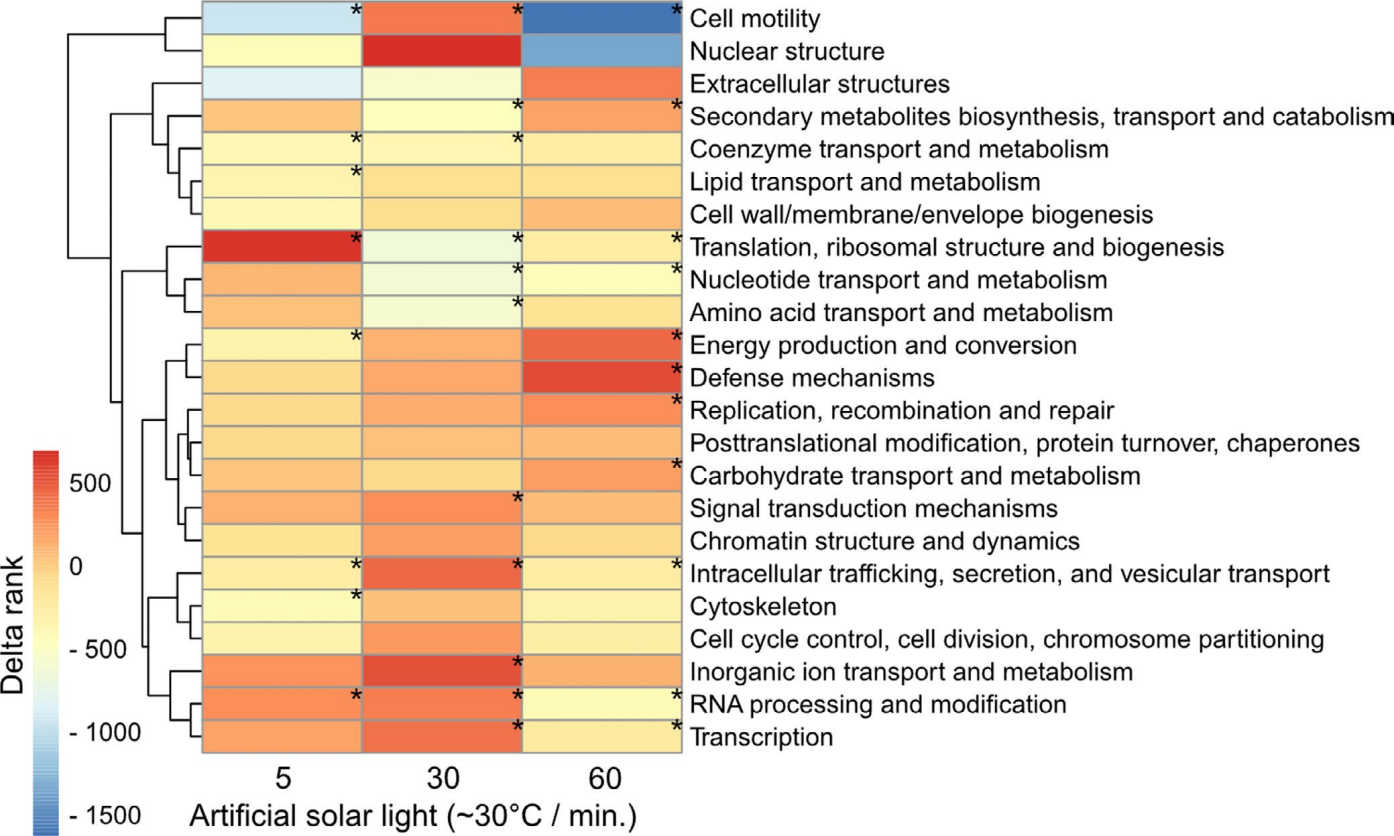
Ranked expression of different KOG classes among the different time points of sun exposure duration. Displayed is the delta rank, which is the difference between the mean rank of genes belonging to this KOG class and all other genes. Darker red or more blue colors indicate whether some KOG classes are significantly enriched with up‐ or downregulated genes. Ranks were calculated using the R package KOGMWU (Dixon *et al*., 2015). Significances were determined using a Mann–Whitney *U* test, using the false discovery method (Benjamini & Hochberg, 1995) and an alpha level of 0.05, indicated by asterisks

To investigate the functional significance of gene expression changes, genes were annotated with KOG classes. Delta ranks (the difference between the mean rank of genes belonging to this KOG class and all other genes) indicate relative upregulation (high delta ranks) or downregulation (low delta ranks) of specific KOG classes in the respective conditions (Figure 3; Table S3). Significant differences in the expression of different KOG classes were detected (Figure 3). The three different time points showed distinct molecular responses to sun exposure. At 5 min of sun exposure, much of the transcriptome is invested in biogenesis (“Translation, ribosomal structure and biogenesis”). As sun exposure continues at 30 min, expression in the mushroom shifts away from the production of biomass, as indicated by significantly reduced expression in the categories “Translation, ribosomal structure and biogenesis,” “Nucleotide transport and metabolism,” and “Amino acid transport and metabolism.” Instead, we found a transient induction of classes of genes that might underlie reconfiguration of the mushroom transcriptome and physiology, including “Signal transduction mechanisms,” “Intracellular trafficking, secretion and vesicle transport,” “Inorganic ion transport and metabolism,” “Transcription,” and “Cell motility.” At 60 min of sun exposure, this response is largely attenuated; expression is enriched for classes of genes involved in mediating stress, for example, “Defense mechanisms,” “Energy production and conversion,” and “Replication, recombination and repair.”

**FIGURE 4:**
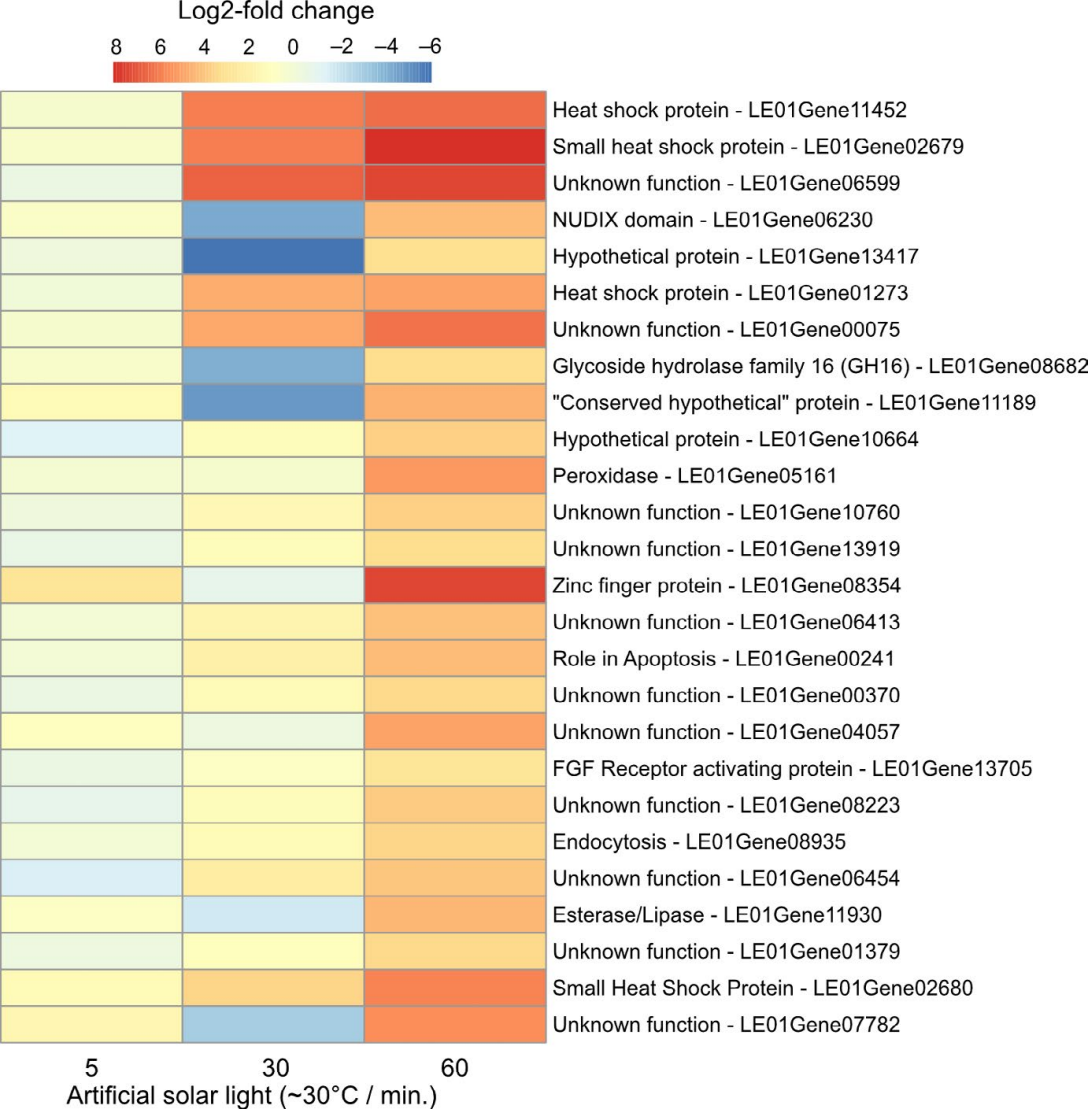
Heatmap of the top 20 most significantly differentially expressed genes after the 60‐min sun exposure. The same genes were added for 5‐ and 30‐min treatment. Functional group annotation was done using EggNOG and BLAST search

Analysis of specific DEGs also provides valuable information regarding responses toward artificial sunlight by mushrooms. We chose to investigate the 20 most significant DEGs for the 60‐min time point. Among the 20 DEGs, four upregulated genes belonged to heat‐shock proteins, which were the highest observed at 60 min (Figure 4). Twelve genes could not be assigned to a specific function and were, thus, classified as “Unknown function” or “Hypothetical protein” (Figure 4). All except one (LG01Gene04057) had homologs in other fungi.

## 4 DISCUSSION

We assessed the molecular responses of the mushroom of *Lentinula edodes* to artificial sun exposure. Sun exposure resembles environmental conditions that increase due to climate change. We found that 30 and 60 min of artificial sun exposure resulted in significant DEGs, whereas 5 min did not yield a significant transcriptional response. KOG class enrichment showed defense mechanisms and upregulation of heat‐shock proteins (HSPs) in the 60‐min treatment. These results suggest that fungi express mushroom‐specific molecular responses toward short‐term sun exposure.

We found that short‐term artificial sun exposure indeed triggered the enrichment or depletion of various DEGs and KOG classes and responses varied with time of artificial sun exposure (Figures 2, 3, 4). Heat‐shock proteins (HSPs) are a molecular marker frequently observed under heat stress conditions in plants, animals, and fungi (Kerner et al., 2012; Tereshina, 2005; Tiwari et al., 2015; Treseder & Lennon, 2015; Zhang et al., 2016). The molecular role of HSPs is to act as chaperones for thermosensitive proteins and prevent degradation (Lindquist & Craig, 1988). Upregulation of HSPs is, thus, a strong indicator for thermally induced molecular responses (Tonsor et al., 2008). These results suggest that fungi and their sexual reproduction structure respond physiologically to varying environmental conditions, the basis to survival under enhanced sun exposure. In a study exposing *Ganoderma lucidum*, a hard‐fleshed mushroom, to heat stress, induction of heat‐shock proteins was found (Zhang et al., 2016). One might not expect such responses in hard‐fleshed fruit bodies because of their trimitic hyphal system, which is assumed to be an adaptation to avoid water loss (Halbwachs et al., 2016). Another study found that heat treatment decreased fresh weight and induced heat‐shock proteins in mushrooms of *Agaricus bisporus* (Lu et al., 2014). The previous studies focused on heat alone, independent from light. Here, we added to this knowledge and showed that also artificial sun exposure induced heat‐shock protein expression. The presence of heat‐shock proteins under heat stress thus seems to be common across fruiting body types. Given that the cited studies manipulated temperature independent from light, we suggest that the heat‐shock protein expression observed in our study is mainly driven by the heating component of our treatment. Increasing tree mortality by climate change will lead to increased canopy openness and thus more sun exposure of mushrooms. Our results suggest mushroom‐specific molecular responses to cope with such harsher conditions. Currently, large‐scale geographical shifts of fungal species based on mushroom surveys have not been observed yet (Andrew et al., 2018; Krah et al., 2019). One explanation might be the ability of the mushrooms to still tolerate changing environmental conditions via molecular responses.

Although our study provides additional insights, it also contains limitations. First, we cannot rule out a systematic response via the mycelium; second, the treatment severity might be low compared with field conditions; and third, only one developmental stage was covered by our study (mature mushrooms): (a) To restrict the sun exposure treatment only to the mushrooms and avoid heating the mycelium, we covered the mushroom using a plant pot (Figures 1 and S1). We cannot completely rule out the possibility that the mycelium was also affected by the sun exposure treatment because the plant pot contained small water rinsing holes. However, it remains to be elucidated whether part of the response we observed was a systematic response, based on the heating of mycelium adjacent to the pot, or signal transduction from the heat‐stressed mushroom to the mycelium. Such systematic responses have been recorded recently when mushroom mycelium was mechanically injured by fungivores (Schmieder et al., 2019). Thus, a sampling of the mycelium as additional control can be helpful to check for such systematic responses. (b) Another limitation of our study might be a lack of treatment severity. Our short‐term artificial solar light treatment was used to mimic sun exposure as occurring under opening canopies due to tree mortality, which is predicted to increase under climate change (Seidl et al., 2014, 2017). We could not observe a severe change in the overall morphology of the mushrooms even at 60 min, indicating that exposure time was too short for inducing morphological defects. Our treatment of 30°C and ca. 100,000 lux over a relatively short period is at the lower range of possible realistic scenarios. One option to increase severity is to use stronger heating lamps. Further, exposure time could be prolonged, and ideally, the fitness loss is quantified, for example, transpiration rate or fresh weight, to demonstrate the severity of the treatment. If, however, longer exposure times are considered, for example, days to weeks, multiple developmental stages of mushroom formation might be affected (Kamada et al., 2010). (c) Our study aimed at testing the effects on the isolated developmental stage of the mature fruit body. However, environmental stress such as sun exposure endure longer than one hour and thus affect mushroom development. Among the *L. edodes* DEGs that were significantly affected by sun exposure (Figure 4), we found no genes that are believed to matter for basidiome developmental morphogenesis of the existing model species (Gupta et al., 2018; Krizsán et al., 2019; Pelkmans et al., 2017). However, it might still be noted that one predicted zinc finger transcription factor (LE08354) was among the most affected DEGs by 60 min of sun exposure treatment (Figure 4). In general, zinc finger transcription factors function as triggers of pivotal (morpho‐)physiological adaptation circuits in various fungal species. For example, zinc finger transcription factors can govern the adaptation of fungi to toxic environmental conditions (e.g., Zcf2, see Hennicke et al., 2013). KOG class assignments imply LE08354 to be involved with protein turnover, post‐translational modification, and chaperones. This, furthermore, supports a putative heat stress‐specific role of the zinc finger transcription factor. Still, zinc finger transcription factors also play a regulatory role in basidiome development (Pelkmans et al., 2017), which might also apply to LE08354. Zinc finger transcription factors might thus be an interesting candidate for functional genetics‐based approaches to study its regulatory role in basidiome development.

Here, we found a mushroom‐specific molecular response to artificial sun exposure; however, it is currently not known whether these responses maintain fungal fitness. Thus, one research avenue for better predictions under climate change is the interspecific differences in the conditions where maturation (spore development) fails and thus fitness decreases. Fungal wood‐inhabiting communities were shown to be strongly influenced by canopy openness (Krah et al., 2018). Thus, one could expect that thermophilic mushroom species have a higher tolerance toward sun exposure than species that preferably grow under closed canopies. Further, since microclimatic change is a continuing process, adaptability across multiple generations might play a role in fungal fitness. Thus, heat‐resistant next to heat‐sensitive species/strains should be the focus of studies testing for heat adaptations such as HSPs and how they help to maintain fruiting capability under stressful conditions.

## 5 CONCLUSIONS

Climate change poses many threats to biodiversity, however, little is known about the ecophysiology of many species under climate change‐induced stress (Scheffers et al., 2016; Urban et al., 2016). This lack of knowledge is especially true for fungi and their mushroom‐specific responses. We found upregulation of heat‐shock protein expression in mushrooms under sun exposure largely independent from the mycelium. These results give first ideas how mushrooms might cope with increased sun exposure, as expected to increase under climate change due to canopy loss, to maintain sexual reproduction success. Nevertheless, in future studies, this understanding of molecular adaptation mechanisms by fungal fruiting structures must be refined, at least by separately investigating the effects of heat and light and by increasing the severity/duration of the abiotic stresses across multiple species.

## CONFLICT OF INTEREST

None declared.

## AUTHOR CONTRIBUTION

**Franz‐Sebastian Krah:** Conceptualization (lead); Investigation (equal); Writing‐original draft (lead). **Jaqueline Hess:** Formal analysis (lead); Writing‐review & editing (equal). **Florian Hennicke:** Formal analysis (supporting); Writing‐review & editing (equal). **Ritwika Kar:** Investigation (equal); Writing‐review & editing (equal). **Claus Bässler:** Conceptualization (support); Resources (lead); Writing‐review & editing (equal).

## Supporting information

Figure S1

Figure S2

Tables S1‐S3

## Data Availability

The datasets generated during and/or analyzed during the current study are available in the DRYAD repository (https://doi.org/10.5061/dryad.mcvdnck1g).
