# Supplementary figures and images for: Transcriptional response of mushrooms to artificial sun exposure
Source: Ecol Evol. 2021 Jul 5;11(15):10538–46. doi: 10.1002/ece3.7862 (PMC8328440; doi:10.1002/ece3.7862)

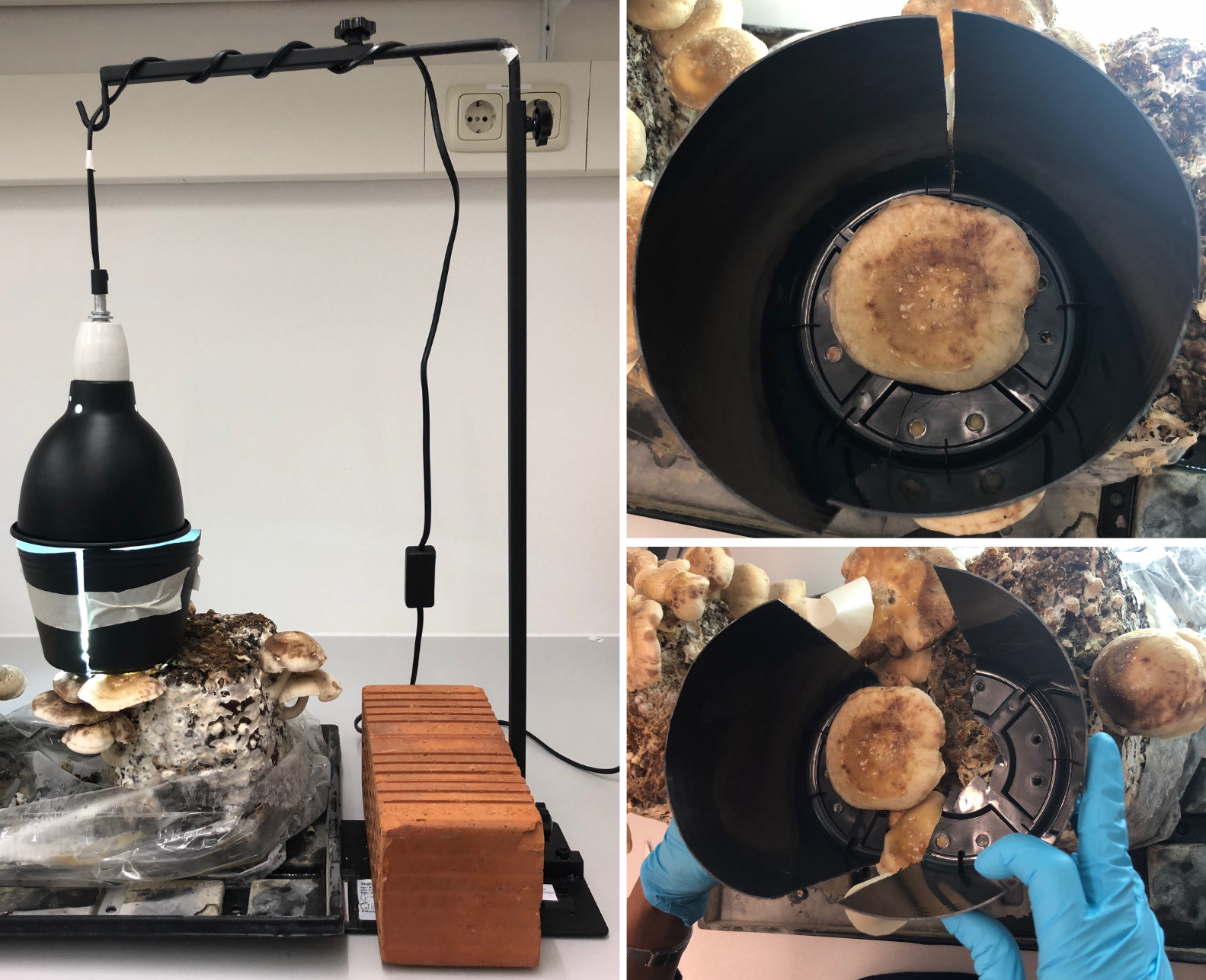

Supplement: Supplementary file 1 — Figure S1 [file ECE3-11-10538-s002.tiff]

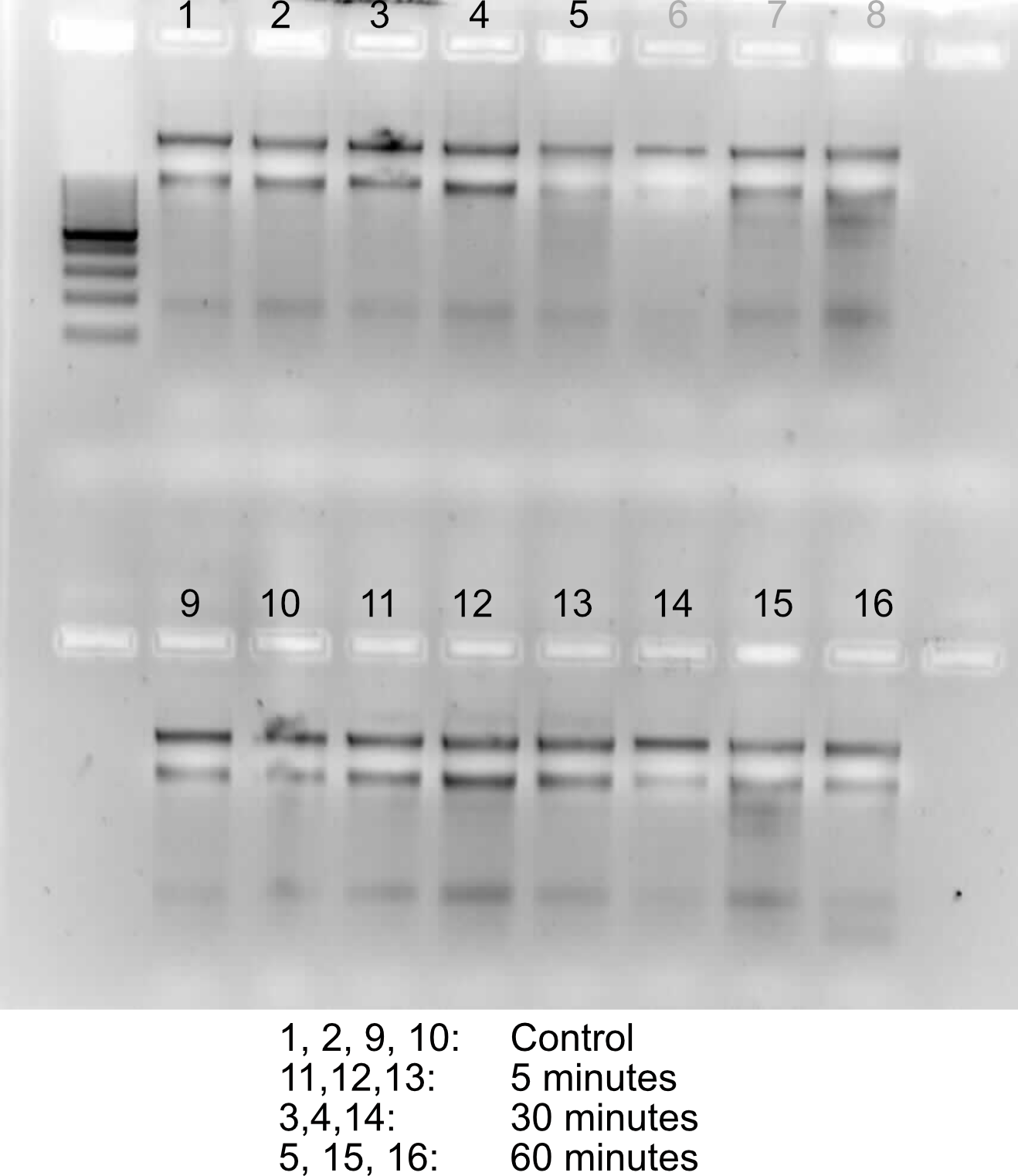

Supplement: Supplementary file 2 — Figure S2 [file ECE3-11-10538-s001.tiff]
