# Supplementary material for: Transcriptional response of mushrooms to artificial sun exposure
Source: Ecol Evol. 2021 Jul 5;11(15):10538–46. doi: 10.1002/ece3.7862 (PMC8328440; doi:10.1002/ece3.7862)
Supplement: Supplementary file 3 — Tables S1‐S3 [file ECE3-11-10538-s003.docx]

**Supplementary material**

**Supplementary tables**

Table S1 RNA quality assessment after RNA extraction. For gel picture, see Fig. S2.

| Sample | 260 Raw | 280 Raw | 320 Raw | 260 | 280 | 260/280 | ng/µL |
| --- | --- | --- | --- | --- | --- | --- | --- |
| 1 | 0.601 | 0.298 | 0.039 | 0.557 | 0.256 | 2.177 | 445.569 |
| 2 | 0.961 | 0.459 | 0.038 | 0.917 | 0.417 | 2.196 | 733.258 |
| 3 | 0.534 | 0.272 | 0.041 | 0.489 | 0.229 | 2.139 | 391.127 |
| 4 | 0.879 | 0.424 | 0.044 | 0.828 | 0.377 | 2.199 | 662.757 |
| 5 | 0.336 | 0.175 | 0.041 | 0.289 | 0.131 | 2.202 | 231.433 |
| 9 | 0.363 | 0.188 | 0.041 | 0.313 | 0.142 | 2.201 | 250.518 |
| 10 | 0.457 | 0.231 | 0.041 | 0.407 | 0.185 | 2.197 | 325.583 |
| 11 | 0.547 | 0.272 | 0.043 | 0.491 | 0.223 | 2.204 | 393.155 |
| 12 | 0.412 | 0.209 | 0.037 | 0.363 | 0.167 | 2.182 | 290.735 |
| 13 | 0.562 | 0.277 | 0.039 | 0.509 | 0.231 | 2.204 | 407.476 |
| 14 | 0.274 | 0.145 | 0.038 | 0.226 | 0.103 | 2.199 | 180.771 |
| 15 | 1.012 | 0.48 | 0.044 | 0.951 | 0.428 | 2.222 | 760.707 |
| 16 | 0.338 | 0.175 | 0.042 | 0.285 | 0.128 | 2.225 | 227.85 |

Table S2 Summary statistics of transcriptome analysis.

| Treatment(minutes) | Block | Total reads | Reads aligned | Mean reads length | Total bases | Bases aligned |
| --- | --- | --- | --- | --- | --- | --- |
| 5 | 2 | 34,118,463 | 94% | 74.43 | 2539.35 | 2384.96 |
| 5 | 2 | 34,271,675 | 96% | 74.44 | 2551.23 | 2438.16 |
| 5 | 2 | 34,091,334 | 95% | 74.47 | 2538.6 | 2421.35 |
| 30 | 1 | 32,447,498 | 97% | 74.46 | 2416.1 | 2333.88 |
| 30 | 1 | 29,891,282 | 95% | 74.46 | 2225.8 | 2108.18 |
| 30 | 1 | 30,287,119 | 95% | 74.45 | 2255.00 | 2137.56 |
| 60 | 2 | 33,933,749 | 93% | 74.45 | 2526.28 | 2344.68 |
| 60 | 2 | 31,199,357 | 93% | 74.43 | 2322.02 | 2166.22 |
| 60 | 2 | 31,973,788 | 95% | 74.40 | 2378.91 | 2250.88 |
| 0 (Control) | 1 | 32,852,074 | 96% | 74.48 | 2446.85 | 2359.65 |
| 0 (Control) | 1 | 33,575,450 | 95% | 74.47 | 2500.37 | 2371.99 |
| 0 (Control) | 2 | 30,843,065 | 95% | 74.48 | 2297.02 | 2180.07 |
| 0 (Control) | 2 | 35,335,967 | 96% | 74.49 | 2632.15 | 2521.32 |

Table S3 KOG enrichment analysis and significance test. Delta ranks above 500 were highlighted in bold. Delta rank is the difference between the mean rank of genes belonging to this KOG class and all other genes.

| Treatment  (minutes) | Term | Nseqs | Delta rank | Pval | Padj |
| --- | --- | --- | --- | --- | --- |
| 5 | Translation. ribosomal structure and biogenesis | 337 | **655** | <0.001 | <0.001 |
| 5 | Lipid transport and metabolism | 291 | -346 | <0.001 | 0.001 |
| 5 | Energy production and conversion | 289 | -304 | 0.001 | 0.005 |
| 5 | RNA processing and modification | 294 | 279 | 0.002 | 0.010 |
| 5 | Intracellular trafficking. secretion. and vesicular transport | 327 | -244 | 0.004 | 0.018 |
| 5 | Cytoskeleton | 85 | -419 | 0.010 | 0.037 |
| 5 | Coenzyme transport and metabolism | 99 | -377 | 0.012 | 0.040 |
| 30 | Translation. ribosomal structure and biogenesis | 337 | -**629** | <0.001 | <0.001 |
| 30 | Amino acid transport and metabolism | 284 | -**576** | <0.001 | <0.001 |
| 30 | Secondary metabolites biosynthesis. transport and catabolism | 433 | -457 | <0.001 | <0.001 |
| 30 | Intracellular trafficking. secretion. and vesicular transport | 327 | 460 | <0.001 | <0.001 |
| 30 | Transcription | 276 | 404 | <0.001 | <0.001 |
| 30 | Inorganic ion transport and metabolism | 139 | **542** | <0.001 | <0.001 |
| 30 | RNA processing and modification | 294 | 359 | <0.001 | <0.001 |
| 30 | Nucleotide transport and metabolism | 89 | -**628** | <0.001 | <0.001 |
| 30 | Signal transduction mechanisms | 385 | 280 | <0.001 | 0.001 |
| 30 | Coenzyme transport and metabolism | 99 | -352 | 0.019 | 0.044 |
| 60 | Energy production and conversion | 289 | 443 | <0.001 | <0.001 |
| 60 | RNA processing and modification | 294 | -404 | <0.001 | <0.001 |
| 60 | Replication. recombination and repair | 349 | 295 | <0.001 | 0.002 |
| 60 | Carbohydrate transport and metabolism | 568 | 213 | 0.001 | 0.007 |
| 60 | Translation. ribosomal structure and biogenesis | 337 | -246 | 0.003 | 0.015 |
| 60 | Intracellular trafficking. secretion. and vesicular transport | 327 | -239 | 0.005 | 0.018 |
| 60 | Defense mechanisms | 52 | **564** | 0.006 | 0.019 |
| 60 | Nucleotide transport and metabolism | 89 | -429 | 0.007 | 0.019 |
| 60 | Secondary metabolites biosynthesis. transport and catabolism | 433 | 196 | 0.008 | 0.021 |
| 60 | Transcription | 276 | -227 | 0.013 | 0.030 |
